# Effects of chronotype on antidepressant treatment and symptoms in patients with depression: a multicenter, parallel, controlled study

**DOI:** 10.1186/s12888-023-04721-z

**Published:** 2023-04-20

**Authors:** Shikai Wang, Min Feng, Yu Fang, Liang Lv, Guilan Sun, Shanfei Cheng, Weiquan Huang, Shengliang Yang, Ping Guo, Mincai Qian, Huanxin Chen

**Affiliations:** 1 Department of Psychiatry, Huzhou Third Municipal Hospital, The Affiliated Hospital of Huzhou University, No. 2088 of Tiaoxi East Road, Wuxing District, 313000 Huzhou, Zhejiang China; 2 Department of Anesthesiology, Huzhou Third Municipal Hospital, The Affiliated Hospital of Huzhou University, 313000 Huzhou, China; 3 Department of Laboratory, Huzhou Third Municipal Hospital, The Affiliated Hospital of Huzhou University, 313000 Huzhou, China

**Keywords:** Depression, Biorhythm, Chronotype, Anhedonia, Antidepressant treatment

## Abstract

**Aim:**

To analyze the changes of chronotypes in patients with depression before and after treatment, and explore the effects of different chronotypes on antidepressant treatment and the dimensions of common symptoms in patients with depression.

**Methods:**

180 patients with depression were selected from 10 tertiary psychiatric hospitals in Zhejiang province, according to the scores of morningness-eveningness questionnaire (MEQ), the patients were divided into three groups: early-type group, middle-type group and late-type group. The 17-item Hamilton Depression Rating Scale (HAMD-17), Hamilton Anxiety Rating Scale anxiety Scale (HAMA), Snaith Hamilton Pleasure Scale (SHAPS), multidimensional fatigue inventory-20(MFI-20) and Pittsburgh sleep quality index (PSQI) were measured at baseline and at the end of the 2nd, 4th, 8th and 12th weeks, the variance analysis of repeated measures was used to analyze the change of each index in the study. The remission rate of depression at each time point was statistically analyzed.

**Results:**

Of the 180 patients included in the study, 26 were lost to follow-up, and 154 were finally included in the analysis. At baseline, 14.93%, 56.5% and 28.57% of the subjects were diagnosed as middle-late type, middle-late type and early-late type respectively, the total scores of Shaps and MFI-20 in the early-type group were higher than those in the late-type group and the middle-type group (*p* < 0.05). During the 12-week antidepressant treatment period, the time effect of PSQI, Shaps, MFI-20 and MEQ had interaction with different time groups (*p* < 0.05). During the treatment, the multiple symptom dimensions of depression were improved to different degrees, but the changing trend was not the same, and the recovery of the anhedonia was obviously delayed, in early-type patients, there are many symptoms such as loss of pleasure and sleep disorders. There was no significant effect on the remission rate of depression in different time type (*p* > 0.05) .

**Conclusion:**

The disorder of chronotypes is common in patients with depression. The time effect of different time type on different symptom dimension of depression was affected, but the effect on remission rate of depression was not significant. To strengthen the management of biological chronotype rhythm in patients with depression may be of great significance in the treatment of depression.

## Introduction

Depression is one of the most common and most important mental diseases in today’s Society [1]. The clinical features of depression are characterized by significant and persistent loss of interest or emotional depression with cognitive, behavioral, physiological, and physical disorders [2]. Depression is the third leading cause of disability worldwide, with a prevalence of 10–77% [3]. Depression is not only a serious mental illness, it is also very harmful to the body. The clinical outcome of 10% of patients with depression is suicide, which not only causes irreparable harm to themselves, but also has a great impact on family and friends [4]. In addition, studies have demonstrated that the presence of depression is associated with increased morbidity and mortality from cerebrovascular disease, cardiovascular disease, and diabetes [5–7]. Although studies on the pathogenesis of depression have shown that it is related to genetics, physiology or biology, little progress has been made in the study of the effect of antidepressant therapy.

Circadian rhythm, also known as the recent day rhythm, refers to life activities to about 24 h as a cycle of changes, day and night alternating physiological function of the human body appeared the corresponding cyclical changes, there are also obvious circadian fluctuations in learning and memory ability, mood and work efficiency [8]. Preliminary studies have shown that biorhythms, especially circadian rhythms, are closely related to the onset, symptom characteristics, comorbidity, prognosis, relapse, and social functioning of depression [9–13]. Among the indicators of circadian disruption, chronotype reflects an individual’s characteristic preference for daily activities and awakenings (morning or evening). According to the individual’s time preference and tendency to organize their own activities in 24-hour daily life, the chronotypes of circadian rhythm can be divided into three different types [14]: early-time type, middle-time type and late-time type. The late-time chronotype, which defines a clear propensity for later sleep and activity habits, has been reported to be associated with delayed circadian phase in patients with mood disorders, and it may be independently associated with emotion regulation, regardless of sleep quality and structure [15].

Chronotype disturbances are prevalent in patients with depression and are an important clinical feature and pathophysiological mechanism of depression, with about half of patients with depression having significant circadian alterations [16].

Due to the late attention to chronotype disorder in depression, there is a lack of attention to the characteristics of biological chronotype disorder and its intervention in clinical practice. Although the link between the circadian system and depression is well established, the biological relationship between chronotype disorder and depression is far from understood, and this study was conducted in conjunction with several large tertiary psychiatric hospitals, a multicenter, parallel and controlled study was conducted to explore the effects of three different time types on the treatment of depression in order to provide new evidence and insights for clinical treatment of depression.

## Materials and methods

### General information

The subjects were selected from 10 psychiatric hospitals in Zhejiang province from March 1,2019 to June 30,2019. The inclusion criteria and exclusion criteria were used as reference, a total of 180 patients were enrolled in the study by a fixed number of consecutive competitive enrollment. At the end of the study, 26 patients were excluded from the analysis due to shedding or incomplete data, and 154 patients were finally included in the study, there were 45 males and 109 females.

Inclusion criteria: (1) patients with depression who met the International Classification of Diseases 10th Revision (ICD-10) diagnostic criteria for depressive episode; (2) MINI-international neuropsychiatric interview (MINI) Chinese version was used for diagnosis screening; (3) patients with acute depressive episode, HAMD-17 ≥ 17, Clinical Global Impression-Severity (CGI-S) ≥ 3; First episode or relapse can be, before 2 weeks without antidepressant treatment and did not receive physical therapy such as convulsive convulsion; (4) All patients had been taking antidepressants for more than six months; (5) Han nationality; gender is not limited, age 18–65 years old; (6) signed informed consent.

Exclusion Criteria: (1) Schizoaffective psychosis, dysthymia or bipolar depression; Any other anxiety disorder as the main diagnosis within 1 year; Substance dependence; personality disorder; (2) Discontinuation of antidepressants or change of medication regimen; (3) Carriers/patients with hepatitis B virus or hepatitis C virus; Patients with abnormal liver function, liver cirrhosis or active liver disease; 3) Patients with severe heart, brain, kidney or endocrine organ diseases or other severe physical diseases; (4) Those who had failed to respond to standard agomelatine therapy in the past, or had failed to respond to adequate treatment with ≥ 2 antidepressants in the current episode; (5) The depressive episode lasted more than 2 years; (6) Had obvious suicide attempt or behavior, and the third item of HAMD (suicide item-RRB- score ≥ 3; 7) Women who are lactating or pregnant, or who require or are unable to use safe and effective contraception during the trial period.

This study was approved and implemented by the Ethics Committee of the Third People’s Hospital of Huzhou City (ethics number: 2019 Ethics Review No. 028). All patients signed informed consent forms.

### Study design

This is an observational study. The demographics characteristics and clinical data of the 154 patients included in the analysis, including age, sex, Body Mass Index (BMI), and whether they had first-episode depression, were collected. The morningness-eveningness Questionnaires (MEQ), Hamilton Depression Scale (HAMD-17), Hamilton Anxiety Rating Scale scale (HAMA), Snaith-Hamilton Pleasure Scale (SHAPS), multidimensional fatigue scale (MFI-20) and Pittsburgh Sleep Quality Index (PSQI) were used to assess the patients at admission. According to the MEQ score, the patients were divided into morning type (early sleep and early wake), Middle Type (general type) and late Type (late sleep and late wake). To evaluate the chronotype disturbance in patients with depression by comparing the baseline data of patients in the three time-type groups. All patients were treated with the same antidepressant for 12 weeks. HAMD-17, HAMA, SHAPS, MFI-20, PSQI and MEQ were assessed at 2,4,8 and 12 weeks after treatment. The therapeutic effect of different types of depression was evaluated by comparing the scores before and after treatment. To analyze the effect of chronotypes on the therapeutic effect of depression, the depression remission rate of each group at each time point was calculated and evaluated. During the study, drug-induced adverse reactions can be treated accordingly. For the drugs used to treat the original somatic diseases before the entry into the group, they can continue to be used in combination. However, during the study period, no combination of antidepressants, anti-anxiety drugs, antipsychotic, mood stabilizers, thyroxines, etc. was allowed, no other systemic psychotherapy, electroconvulsive therapy or other physical therapy were used in combination. Depending on the patient’s condition, a short-term combination of low-dose benzodiazepine or zolpidem may be used, but not for more than 1 week, so as not to interfere with the observation and analysis of the patient’s inherent chronotype rhythm.

### Research methodology

The mental state scale was carried out by 2 doctors with intermediate or higher professional title in each center, and each patient was assessed with cross-blinding method without knowing their grouping.

MEQ is commonly used to assess chronotypes [17]. There are 19 items in this questionnaire. Each item is scored on a scale of 0 to 6, with a total score of 16 to 86. On the basis of these scores, individuals were classified as early type (score 59–86), middle type (score 42–58), or Late type (score 16–41) .

SHAPS is a measure of pleasurable experience [18], which consists of 14 items covering interest/entertainment, social interaction, sensory experience, and diet, the subjects were asked to give 1–4 marks to the items listed respectively, and the total score of the scale was 14–56. The higher the total score, the more serious the degree of anhedonia.

MFI-20 includes General Fatigue, Physical Fatigue, Reduced Activity, Reduced Motivation, and Mental Fatigue on a scale of 20 to 100 across five dimensions, the higher the score, the higher the degree of fatigue [19].

PSQI is used to assess sleep quality in subjects [20]. The PSQI consists of 19 self-rated items and five other rated items, there are 7 factors: sleep quality, sleep time, sleep time, sleep efficiency, sleep disorder, hypnotic drugs and daytime dysfunction. The scores of each factor ranged from 0 to 3, and the total score ranged from 0 to 21, with 7 being the cut-off value of sleep quality. The higher the score, the worse the sleep quality.

HAMA is used to assess the severity of anxiety symptoms [21]. Hama has 14 items, using a five-grade score of 0–4: (0) asymptomatic, (1) mild, (2) moderate, (3) severe, (4) extremely severe. A total score of ≥ 29 indicates severe anxiety; a total score of ≥ 21 indicates definite anxiety; a total score of ≥ 14 indicates definite anxiety; a total score of ≥ 7 indicates possible anxiety; and a total score of < 7 indicates no symptoms of anxiety.

HAMD-17 is a 17-item replacement scale [22]. Each question has a score of 0–2 or 0–4, with a total score of 0–52. The higher the total score, the more severe the depression. A score of 0–7 indicates no depression, a score of 8–17 indicates mild depression, a score of 18–24 indicates moderate depression, and a score of 25–52 indicates severe depression.

### Outcome measures

HAMD-17 and HAMA were assessed at baseline and at the end of the 2nd, 4th, 8th and 12th weeks, and SHAPS, MFI-20, MEQ and PSQI were assessed. During the Treatment, the complaints and adverse reactions were recorded and evaluated by Treatment Emergent Symptom Scale (TESS) .

The effective remission rate was defined as the reduction rate of the total score of HAMD-17 > 50% before and after treatment, and the clinical cure was defined as the total score of HAMD-17 ≤ 7.

### Statistical analysis

SPSS 22.0 software was used for data processing. For the measurement data, the mean ± standard deviation was used for normal distribution, and one-way analysis of variance was used for comparison of differences between groups. For categorical variables or count data, frequencies (percentages) were used, and categorical variables were compared using the Chi-square (Χ^2^) test or Fisher’s exact test. The test level α is 0.05. P < 0.05 indicated that the difference was statistically significant.

## Results

### Baseline characteristics of patients in each group

Of the 180 patients with depression enrolled, 26 (14.45%) were not included in the analysis because of shedding or incomplete data, and 154 (85.55%) were ultimately included in the analysis, including 45 men and 109 women. According to the MEQ score, 154 patients were divided into 3 groups, 23 (14.93%) in the late-type group, 87(56.5%) in the intermediate-type group, and 44 (28.57%) in the early-type group. Baseline characteristics of patients in each group are shown in Table 1. At baseline, the age of patients with early-type depression was significantly higher than that of patients with intermediate-type and late-type depression (*p* < 0.05), suggesting that age may affect the chronotypes of patients. The total scores of Shaps and MFI-20 in the early depression group were significantly higher than those in the late depression group and the middle depression group (*p* < 0.05), this suggests that the degree of pleasure loss and fatigue may be greatly affected by chronotype. In addition, there were no significant differences in sleep quality, anxiety and depression scores among the three groups (*p* > 0.05), and there were no significant differences in gender, BMI, relapse and antidepressant use (*p* > 0.05) .

**Table 1:** Baseline demographic data and clinical assessment of patients with depression in each group

Items	Late-type group (n = 23)	Middle-type group (n = 87)	Early-type group (n = 44)	P value
Sex				
Male (n, %)	6 (13.33)	27(60)	12(26.67)	0.849
Female (n, %)	17 (15.69)	60(55.05)	32(29.36)	
Age	40.22 ± 14.65	41.85 ± 14.23	48.20 ± 9.18	0.016
BMI	22.19 ± 2.54	21.31 ± 2.97	22.10 ± 3.09	0.232
First episode/Relapse				
First episode (n, %)	12 (13.79)	46 (52.88)	29 (33.3)	0.329
Relapse (n, %)	11 (16.42)	41 (61.19)	15 (22.39)	
Antidepressant use (mg)				
Fluoxetine	35.86 ± 7.24 (n = 2)	33.38 ± 12.16 (n = 6)	32.64 ± 11.42 (n = 3)	0.864
Paroxetine	28.94 ± 16.44 (n = 3)	31.72 ± 18.57 (n = 10)	29.62 ± 16.28 (n = 5)	
Sertraline	164.32 ± 26.16 (n = 5)	152.84 ± 38.81 (n = 16)	143.72 ± 32.42 (n = 8)	
Citalopram	35.12 ± 11.04 (n = 2)	35.62 ± 13.44 (n = 6)	34.87 ± 14.36 (n = 4)	
Escitalopram	13.72 ± 4.47 (n = 3)	12.64 ± 3.32 (n = 14)	13.16 ± 5.44 (n = 6)	
Agomelatine	36.22 ± 11.42 (n = 8)	32.36 ± 9.47 (n = 35)	33.34 ± 6.28 (n = 18)	
HAMD_− 17_	21.61 ± 8.16	23.02 ± 5.66	22.07 ± 3.92	0.466
HAMA	19.61 ± 9.44	21.16 ± 9.36	21.30 ± 5.09	0.595
MEQ	37.96 ± 2.87	50.02 ± 4.51	64.91 ± 3.85	< 0.0001
PSQI	14.91 ± 2.21	14.23 ± 3.54	14.55 ± 3.59	0.666
SHAPS	44.87 ± 4.35	46.64 ± 6.92	43.57 ± 2.19	0.012
MFI-20	81.82 ± 8.41	75.47 ± 12.50	85.34 ± 10.13	< 0.0001

### The changes of the core symptoms and biological rhythm of the depressive patients in each group were analyzed

**Fig. 1:**
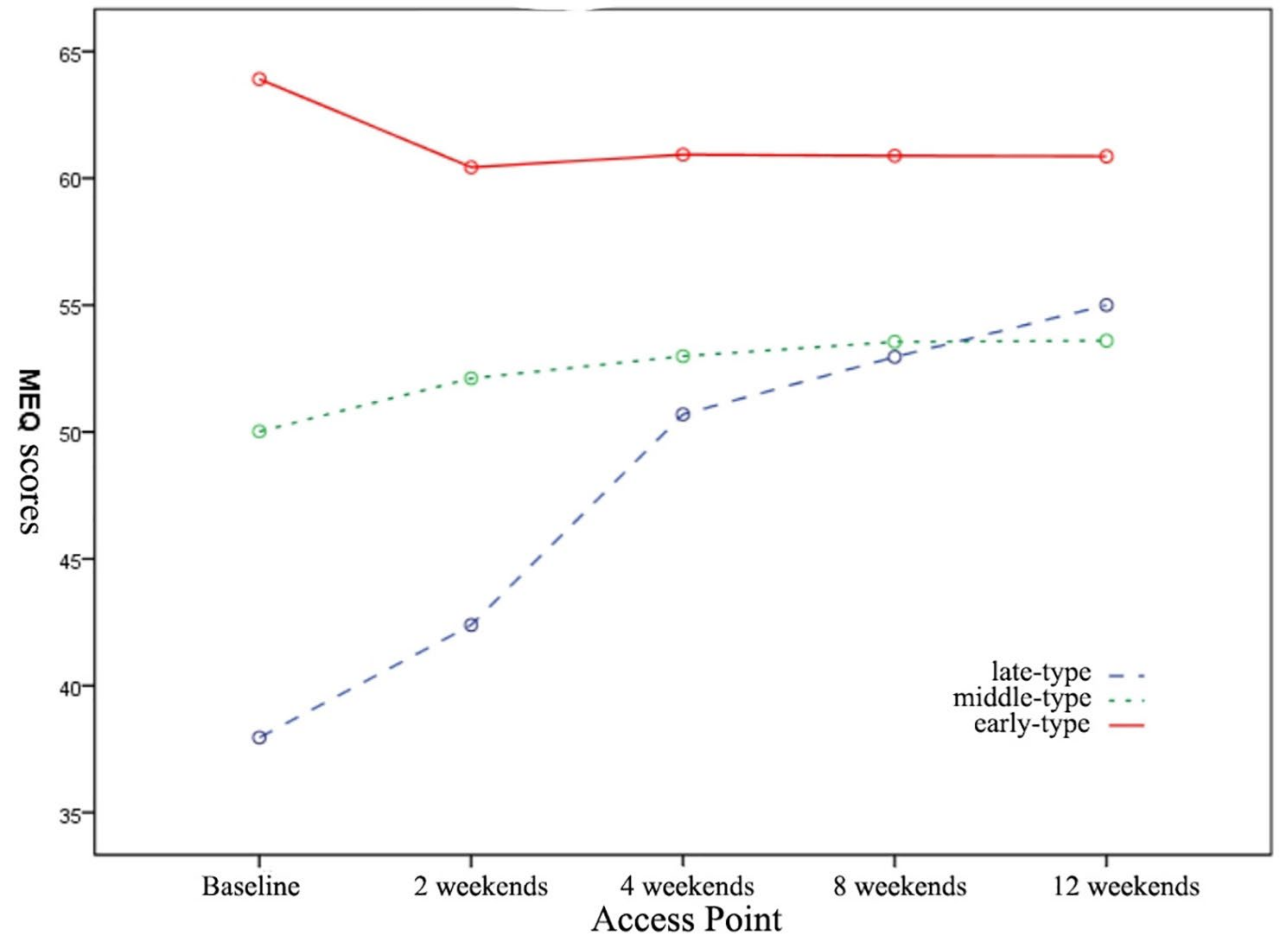
Changes of MEQ scores at different visit points in patients with different types of depression

**Fig. 2:**
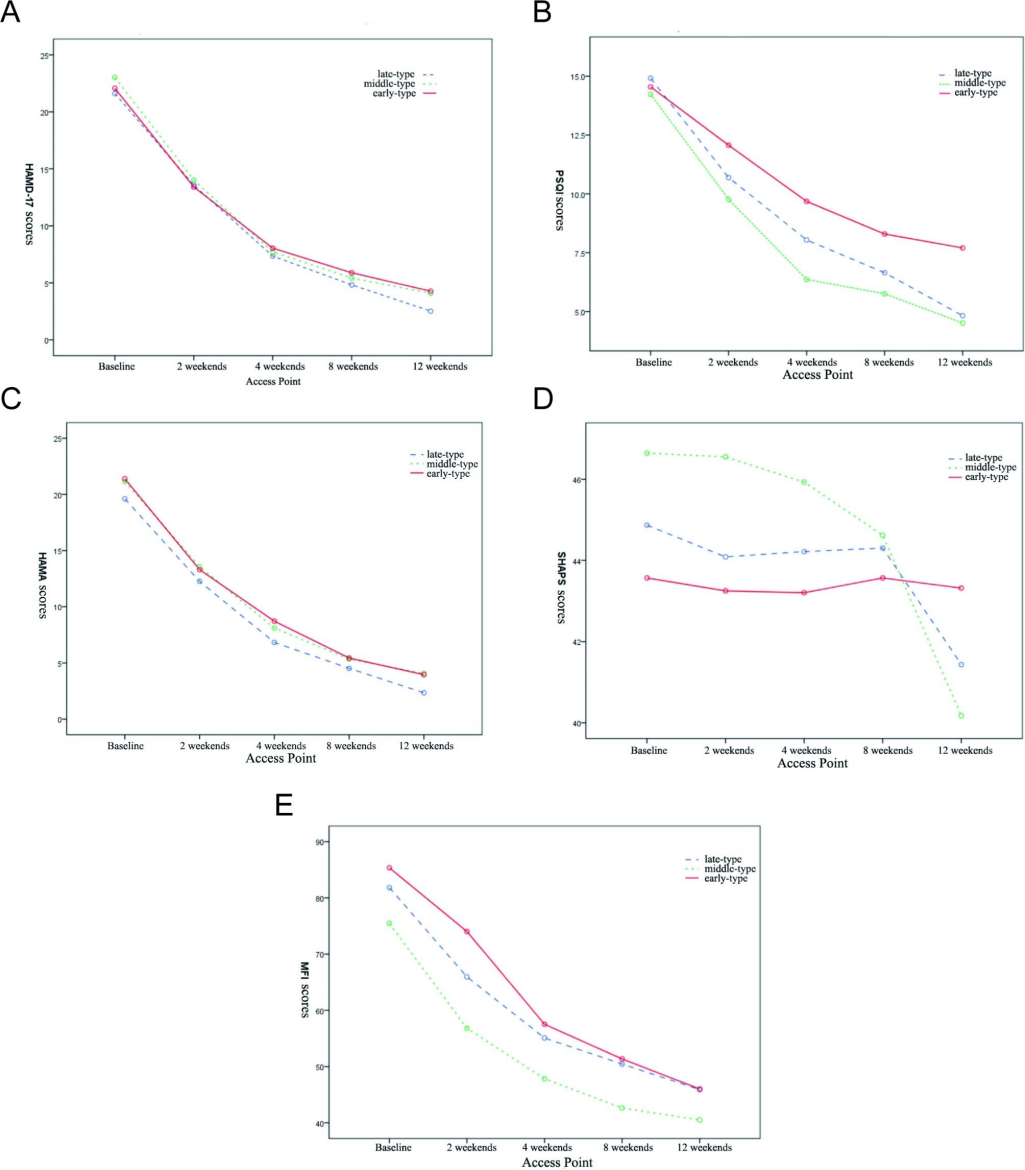
**A.** Changes of HAMD-17 scores at different visiting points in patients with different types of depression; **B.** Changes of PSQI scores at different visit points in patients with different types of depression; **C.** HAMA scores of depression patients at different time points of visit; **D.** Changes of SHAPS scores at different visit points in patients with different types of depression; **E.** The changes of MFI score at different visit points in patients with different types of depression

The MEQ scores of the three groups showed different trends of change. The MEQ scores of the early-type group showed a decreasing trend, while the MEQ scores of the middle-type group increased relatively slowly, while the late-type group showed a significant increase (Fig. 1). The scores of HAMD-17, PSQI, HAMA, SHAPS and MFI-20 were decreased in all three groups during the 12-week antidepressant treatment, the time effect of each group was significant (*p* < 0.01) (Table 2; Fig. 2A-E). There were significant differences among the three groups except HAMD-17 and HAMA (*p* < 0.05). In addition, except HAMD-17 and HAMA, the scores of other scales in the three groups had interaction between treatment time and different time types (*p* < 0.001, Fig. 2B, D and E), this suggests that the time-effect of antidepressant treatment varies across time-dependent patient populations (Table 2) .

**Table 2:** The changes of depression-related scale scores in each group

Items	Group	Case	Access Point					F value and p value
			0wk	2wk	4wk	8wk	12wk	
MEQ	late-type group	23	37.96 ± 2.87	42.39 ± 6.60	50.70 ± 7.24	52.96 ± 7.80	55.00 ± 8.20	F_T_=13.888, P<0.001 F_I_=9.669, P<0.001 F_T*I_=43.828, P<0.001
	middle-type group	87	50.02 ± 4.51	52.11 ± 6.24	52.99 ± 8.09	53.55 ± 9.15	53.60 ± 10.05	
	early-type group	44	64.91 ± 3.85	60.43 ± 6.49	60.93 ± 8.96	60.89 ± 9.55	60.86 ± 9.42	
HAMD-17	late-type group	23	21.61 ± 8.16	13.57 ± 10.30	7.35 ± 7.91	4.83 ± 4.93	2.52 ± 2.75	F_T_=479.759, P<0.001 F_I_=0.619, P = 0.706 F_T*I_=0.352, P = 0.407
	middle-type group	87	23.02 ± 5.66	14.01 ± 7.33	7.72 ± 6.17	5.43 ± 5.84	4.09 ± 4.15	
	early-type group	44	22.07 ± 3.92	13.41 ± 4.53	8.05 ± 3.61	5.89 ± 2.66	4.27 ± 2.68	
HAMA	late-type group	23	19.61 ± 9.44	12.26 ± 9.41	6.83 ± 7.80	4.52 ± 5.15	2.35 ± 2.39	F_T_=319.528, P<0.001 F_I_=0.156, P = 0.996 F_T*I_=0.640, P = 0.529
	middle-type group	87	21.16 ± 9.36	13.56 ± 8.84	8.11 ± 7.51	5.36 ± 5.01	4.07 ± 4.68	
	early-type group	44	21.30 ± 5.09	13.07 ± 4.51	8.64 ± 4.08	5.48 ± 2.54	3.95 ± 3.50	
PQSI	late-type group	23	14.91 ± 2.21	10.70 ± 3.04	8.04 ± 3.48	6.65 ± 3.24	4.83 ± 2.76	F_T_=141.134, P<0.001 F_I_=2.858, P = 0.004 F_T*I_=8.916, P<0.001
	middle-type group	87	14.23 ± 3.54	9.77 ± 4.38	6.37 ± 4.22	5.76 ± 3.99	4.52 ± 3.37	
	early-type group	44	14.55 ± 3.58	12.07 ± 4.44	9.68 ± 3.63	8.30 ± 3.32	7.70 ± 3.14	
SHAPS	late-type group	23	44.87 ± 4.35	44.09 ± 4.11	44.22 ± 3.36	44.30 ± 3.75	41.43 ± 4.27	F_T_=3.629, P = 0.007 F_I_=2.193, P = 0.028 F_T*I_=2.898, P = 0.058
	middle-type group	87	46.64 ± 6.92	46.55 ± 6.63	45.93 ± 6.25	44.62 ± 5.71	40.17 ± 5.79	
	early-type group	44	43.57 ± 2.19	43.25 ± 2.88	43.20 ± 2.74	43.57 ± 3.69	43.32 ± 3.97	
MFI-20	late-type group	23	81.82 ± 8.41	65.61 ± 15.05	54.61 ± 13.58	50.91 ± 10.87	46.61 ± 10.82	F_T_=94.316, P<0.001 F_I_=1.982, P = 0.048 F_T*I_=16.579, P<0.001
	middle-type group	87	75.47 ± 12.50	56.82 ± 16.05	47.84 ± 16.85	42.63 ± 14.55	40.52 ± 16.37	
	early-type group	44	85.34 ± 10.13	74.02 ± 15.77	57.52 ± 12.58	51.36 ± 13.90	46.02 ± 12.12	

F_T_: Test of significance of difference in different time points

F_I_: Significance test of the difference among the three groups

F_T*I_: Test of interaction significance between different scales and different types of time

### The remission rate of depressive symptoms in the three groups at the end of the study

The effective remission rate of the total score of Hamd-17 was more than 50% before and after treatment, and the clinical cure rate of the total score of HAMD-17 ≤ 7 was calculated. Results as shown in Table 3, the remission rate of depressive symptoms in all three groups increased in turn during antidepressant treatment (*p* < 0.05 or *p* < 0.001). The remission rate of late-onset depression was higher than that of the other two groups during the study, but there was no significant difference in remission rate among the three groups (*p* > 0.05) .

**Table 3:** comparison of remission rates among the three groups after antidepressant treatment

Group	Case	Baseline	2weeks	4 weeks	8 weeks	12 weekends	χ^2^	P
late-type group	23	0	8 (34.78%)	9 (39.13%)	15 (65.22%)	19 (82.61%)	14.211	0.003
middle-type group	87	0	15 (17.24%)	22 (25.29%)	55 (63.22%)	69 (79.31%)	93.152	< 0.001
early-type group	44	0	6 (13.64%)	11 (25%)	27 (61.36%)	34 (77.27%)	47.983	< 0.001
			χ^2^ = 4749 P = 0.093	χ^2^ = 1.918 P = 0.383	χ^2^ = 0.101 P = 0.951	χ^2^ = 0.262 P = 0.877		

## Discussion

The change of sleep chronotype in depressive patients has become a new perspective to explore the occurrence and development of depression. This multicenter study of the Chinese population provides further evidence of the relationship between chronotypes and the efficacy of antidepressant therapy.

Previous surveys have shown that among the general population aged 30–50 years, the detection rates of late-type, intermediate-type and early-type are 5.6%, 44.6% and 49.8%, respectively [23]. In our study, 14.93%, 56.5%, and 28.57% of the depression patients included in the analysis were found to have these temporal patterns at baseline, indicating that compared with the normal population, patients with depression have obvious symptoms of abnormal circadian rhythm. Many non-clinical samples showed that night type was positively correlated with depressive symptoms, and had a positive predictive effect on depressive symptoms and depression, while morning type had the opposite effect. De Souza and Hidalgo [24] found that the later the midpoint of sleep on weekdays and days off (tending to be nighttime), the more severe the depressive symptoms were, and the later midpoint of sleep had a positive predictive effect on mild depressive symptoms (BDI ≥ 10). A logistic regression analysis by Merikanto et al. [25] showed that the late-onset type was significantly to be diagnosed with depression, antidepressant use, and depressive symptoms than intermediate and early-onset types. In the study of clinical samples also showed that night-type has a significant positive predictive effect on depression [26, 27].

Interestingly, in this study, the total scores of PSQI, Hama and Hamd-17 were not significantly different among the different time types. The MFI-20 total scores were higher in the early time type patients, while the Shaps total scores were higher in the middle time type patients, in other words, the severity of sleep quality, anxiety symptoms and depressive symptoms were similar in the patients with dysrhythmia, but the severity of different symptom dimensions might be different in the patients with different chronotypes. This may be due to the differences of the subjects, measurement tools and the different division of the factor structure of sleep duration, which have an impact on the relationship between sleep duration and depression. For example, Furusawa et al. [28] showed that the risk of developing a depressive state (SDS ≥ 45) in the intermediate type was 1.67-fold higher than that in the early type in their logistic regression analysis of the Japanese shift workers survey using the Self-rating Depression Scale (SDS); The incidence of late-onset depression was not significantly different (OR = 3, *p* = 0.074), which may be due to the small number of late-onset depression (N = 20,2.3%) .

Over the course of the 12-week study, the total MEQ scores of patients with different types of depression gradually increased in our study. But in addition, the total scores of HAMD-17, HAMA, SHAPS, MFI-20 and PSQI decreased in turn, and the time effect in each group was significant (*p* < 0.01), common concomitant symptoms such as anxiety, insomnia and dysrhythmia can also be improved by treatment, which has been reported in the past [29]. However, the change trend of different scales was different among the three groups, which could be roughly divided into three types: ① the total scores of HAMD-17 and Hama improved and the trend of change was the same during the study period; There was no significant difference among the three groups at each visit point. ② in fatigue and sleep quality, the total scores of MFI-20 and PSQI improved during the study period (*p* < 0.01) and the trend of change was basically the same, but there were significant differences among the three groups at each visit point, the severity of symptoms was early type > late type > middle type, which suggested that the patients with early type might have early awakening and low-dynamic symptoms. ③ there were significant differences in Shaps and MEQ among the three groups in terms of loss of pleasure and rhythm disturbance, and Meq showed the most obvious differentiation trend with time, the MEQ scores of early-type patients decreased within 2 weeks and leveled off thereafter, while those of late-type patients showed a continuous increasing trend throughout the study period and maintained until the end of the study, the MEQ scores of patients with intermediate-time depression did not change significantly during the study period. The SHAPS score changed slowly in general, indicating that the improvement of the symptoms of anhedonia lagged significantly behind the relief of other symptoms. The patients with intermediate and late anhedonia showed significant improvement after the 8th week of treatment, however, the loss of pleasure in patients with early-onset depression did not improve significantly throughout the study, even at the end of the study was also common residue. It is suggested that patients with early-onset depression at baseline may have residual symptoms of anhedonia.

Studies of clinical samples have shown that late-onset antidepressants have a poor prognosis, and late-onset patients had fewer depression scores after the intervention compared with early-onset and intermediate-onset patients [30]. A longitudinal study of 253 patients with major depressive disorder by Chan et al. [31] found a 3-fold higher risk of unrelieved late-onset major depression than non-late-onset major depression after 6 years. However, a follow-up study of patients with depressive disorders by Druiven et al. [32] found that sleep duration did not predict remission or maintenance of depressive disorders. Consistently, in this study, patients with different types of depression showed a trend of increasing remission rates at each point of visit during the study (p < 0.01), however, there was no significant difference among the three types of chronotypes at each visit point (*p* > 0.05), which suggested that different chronotypes had no significant effect on the remission rate of depressive symptoms.

There are some limitations in this study. First of all, this study measured sleep chronotype by self-reported questionnaire total score or midpoint of sleep, which essentially regarded sleep chronotype as a single-dimensional structure, and sleep chronotype may be a multidimensional structure composed of different components.

Secondly, the current research is still focused on the relationship between sleep duration and depression, the internal mechanism of the relationship between the two is less studied, the future study will enrich the internal mechanism of the relationship between sleep duration and depression. Third, this study on the relationship between sleep patterns and depression to the observational research-based, related to the intervention study is still less, the future will strengthen the intervention of empirical research.

## Conclusion

In the course of antidepressant treatment, sleep chronotypes had great effects on fatigue, loss of pleasure and sleep quality in the dimensions of depressive symptoms, but the effect on remission rate of depression was not significant. It may be important for the treatment of depression to strengthen the regulation of biological chronotype in patients with depression.

## Data Availability

All data generated or analyzed during this study are included in this published article.
